# A comparison of the metabolic side-effects of the second-generation antipsychotic drugs risperidone and paliperidone in animal models

**DOI:** 10.1371/journal.pone.0246211

**Published:** 2021-01-28

**Authors:** Heidi N. Boyda, Ric M. Procyshyn, Lurdes Tse, Jessica W. Y. Yuen, William G. Honer, Alasdair M. Barr

**Affiliations:** 1 Department of Anesthesiology, Pharmacology & Therapeutics, University of British Columbia, Vancouver, Canada; 2 Department of Psychiatry, University of British Columbia, Vancouver, Canada; 3 British Columbia Mental Health & Addictions Research Institute, Vancouver, Canada; Wayne State University, UNITED STATES

## Abstract

**Background:**

The second generation antipsychotic drugs represent the most common form of pharmacotherapy for schizophrenia disorders. It is now well established that most of the second generation drugs cause metabolic side-effects. Risperidone and its active metabolite paliperidone (9-hydroxyrisperidone) are two commonly used antipsychotic drugs with moderate metabolic liability. However, there is a dearth of preclinical data that directly compares the metabolic effects of these two drugs, using sophisticated experimental procedures. The goal of the present study was to compare metabolic effects for each drug versus control animals.

**Methods:**

Adult female rats were acutely treated with either risperidone (0.1, 0.5, 1, 2, 6 mg/kg), paliperidone (0.1, 0.5, 1, 2, 6 mg/kg) or vehicle and subjected to the glucose tolerance test; plasma was collected to measure insulin levels to measure insulin resistance with HOMA-IR. Separate groups of rats were treated with either risperidone (1, 6 mg/kg), paliperidone (1, 6 mg/kg) or vehicle, and subjected to the hyperinsulinemic euglycemic clamp.

**Results:**

Fasting glucose levels were increased by all but the lowest dose of risperidone, but only with the highest dose of paliperidone. HOMA-IR increased for both drugs with all but the lowest dose, while the three highest doses decreased glucose tolerance for both drugs. Risperidone and paliperidone both exhibited dose-dependent decreases in the glucose infusion rate in the clamp, reflecting pronounced insulin resistance.

**Conclusions:**

In preclinical models, both risperidone and paliperidone exhibited notable metabolic side-effects that were dose-dependent. Differences between the two were modest, and most notable as effects on fasting glucose.

## Introduction

Antipsychotic medications are prescribed for the treatment of an increasing number of psychiatric disorders, as their clinical use has extended beyond their original target of schizophrenia spectrum disorders [1–4]. They are usually categorized into two main classes, which include the first generation drugs (also known as “typical antipsychotics”) that were developed from the mid-1950s, and the second generation antipsychotics (also known as “atypical antipsychotics”) that came to market in the 1980s. The second generation drugs are largely preferred to their predecessors due to their lower incidence of neurological side-effects, which include extrapyramidal symptoms and tardive dyskinesia [5]. However, after several decades of use, it is well established that the second generation drugs have their own side-effects, which predominantly include metabolic dysregulation, characterized by weight gain, hyperglycemia, hyperinsulinemia and hyperlipidemia [6–12]. These side-effects can ultimately lead to an increased risk of developing cardiometabolic disorders such as Type 2 diabetes mellitus (DM) [13,14]. Importantly, the metabolic side-effects of second generation antipsychotics range along a spectrum, depending on the specific drug [15].

The second generation antipsychotic drug risperidone remains one of the most frequently used antipsychotic drugs worldwide [2,16]. The drug’s popularity among prescribers is based on a large body of evidence supporting its clinical efficacy and ease of use, with minimal need for monitoring, while dosing options are suitable for many off-label indications in youth and the elderly [17–20]. In terms of metabolic liability, the drug remains somewhere in the middle of the class, with fewer and less severe metabolic side-effects than olanzapine and clozapine, but greater than some of the newer second generation drugs, such as asenapine and lurasidone [21–23]. Paliperidone (9-hydroxyrisperidone), which represents the active metabolite of risperidone, is a more recent addition to the second generation class of antipsychotic drugs [24]. Despite its similarity to the parent compound, paliperidone displays differences to risperidone in terms of its affinity for various central and peripheral receptors [25], including adrenoceptors as well as receptors for other monoamines (including dopamine and serotonin). Many of these receptors are believed to contribute to the acute metabolic effects of antipsychotic drugs [26–28]. There are also important pharmacokinetic differences between both drugs, in terms of metabolism via liver enzymes and transport into the brain by the transporter P-glycoprotein [29]. All of these differences may affect the metabolic liability of the compounds.

Assessing the acute metabolic effects of antipsychotic drugs in patients can be challenging, as metabolic dysregulation can be caused by many unrelated variables such as smoking and alcohol use, poor diet and lack of exercise [30,31], and invasive procedures may not be well-tolerated. To this end, animal models of antipsychotic-induced metabolic side-effects have been helpful and informative in characterizing the acute metabolic effects of antipsychotics [32]. These studies have confirmed that treatment with most second generation antipsychotics can cause glucose intolerance and insulin resistance, often within minutes [33–44]–effects that have been confirmed in non-psychiatric human subjects [45–47] including in a recent meta-analysis [48].

In current clinical practice, both paliperidone and the parent drug risperidone are often prescribed interchangeably [49], as they have a similar therapeutic profile and both exist in depot formulations [50–52]. However, the acute metabolic effects of the two drugs in comparison to each other are not well characterized in the preclinical literature. Indeed, there is minimal information about the metabolic effects of paliperidone in animal models. Given that even modest differences in metabolic liability can result in large cumulative effects on physical health over time [53], it is important to know whether the two antipsychotics differ in their metabolic dysregulation. The goal of the present study was therefore to compare the metabolic effects of the two drugs directly, across a range of different doses of each drug, using the glucose tolerance test to measure glucose intolerance, and the “gold standard” hyperinsulinemic-euglycemic clamp to measure insulin resistance.

## Methods

### Animals

Adult female Sprague-Dawley rats (250–275 g) were purchased from the animal supplier (Charles River, Montreal, QC) and habituated to the UBC colony for at least one week prior to the commencement of experiments. Females are used by our laboratory and many other groups because they exhibit more consistent metabolic abnormalities than males [32,33,54,55]. Rats were pair-housed and maintained on a 12-hour light-dark cycle (lights on at 07:00 hours) with ambient temperature (22 ± 1°C), and food and water available *ad libitum*. All experimental procedures were approved by the UBC Animal Care and Use Committee and animals were treated in accordance with the NIH Guidelines for the Care and Use of Laboratory Animals.

### Pharmacological agents and solutions

The antipsychotic drugs risperidone and paliperidone [Toronto Research Chemicals Inc., Toronto, ON] were prepared fresh daily. Both were formulated in 50% polyethylene glycol 400, 40% distilled water and 10% ethanol (PEG solution) as per previously [56,57]. The antipsychotics were dissolved in a volume of 1 ml/kg for subcutaneous (SC) injection. A previous study noted that acute administration of risperidone by SC injection resulted in peak plasma levels of the drug between 0.5–1 hr after administration, with a plasma elimination half-life of approximately 1 hr [58], meaning that drug levels of both compounds were high during tolerance test and clamp procedures. For the clamp experiments, recombinant human insulin (Humulin R) [Eli Lily, Indianapolis, IN) and dextrose (50%) were formulated in 0.9% w/v saline. All other chemicals were of reagent grade.

**Fig 1:**
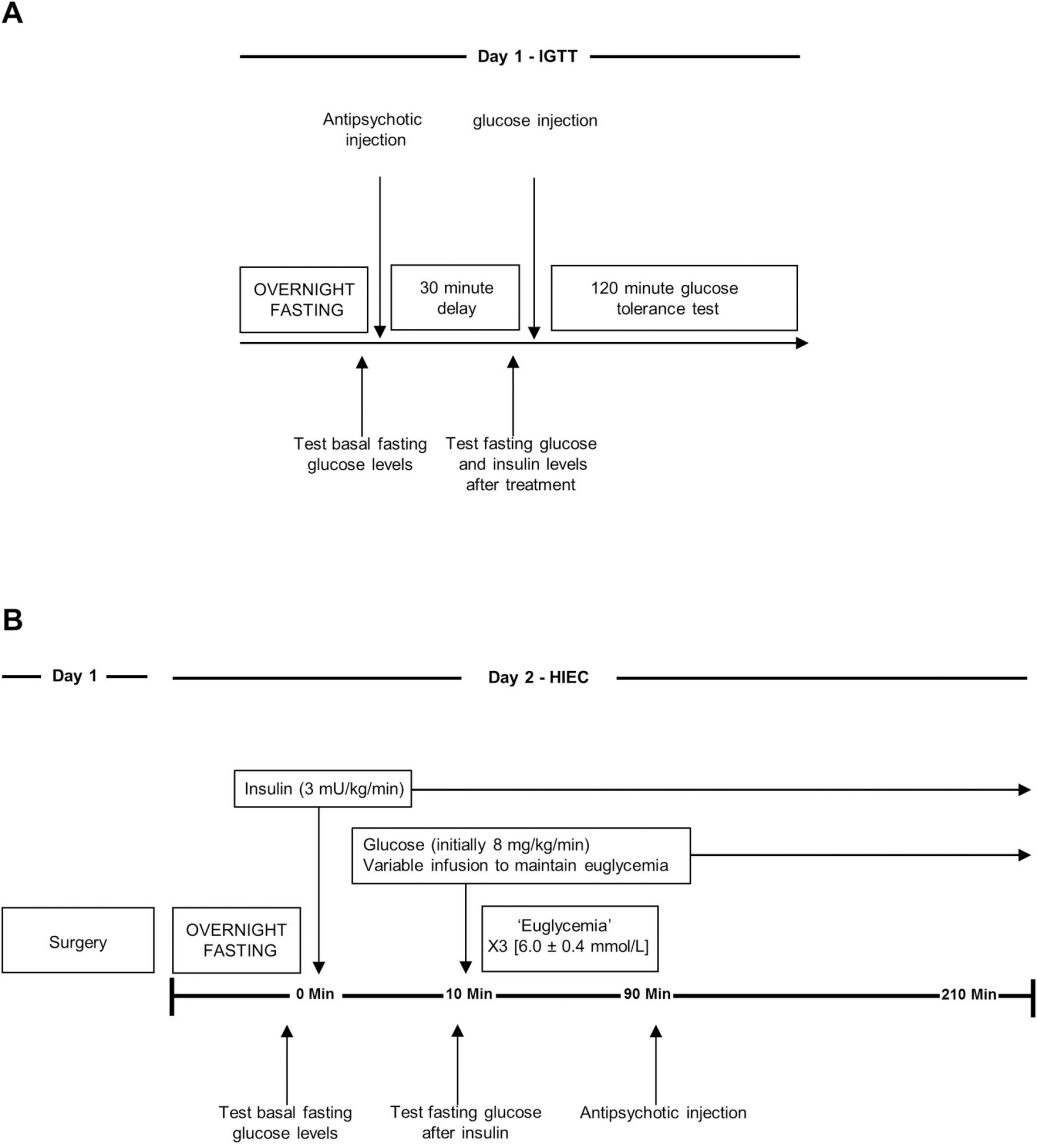
Sequence of experimental procedures. Figure graphically illustrates (**A**) intraperitoneal glucose tolerance test (IGTT) and (**B**) hyperinsulinemic-euglycemic clamp (HIEC) in which the two antipsychotic drugs risperidone and paliperidone are administered *in vivo*.

*Intraperitoneal Glucose Tolerance Test (IGTT)* (see Fig 1A for sequence of experimental procedures). Rats were fasted overnight (16 ± 2 hours) and randomly assigned to one of five treatment groups for each drug: risperidone (0.1, 0.5, 1, 2 and 6 mg/kg s.c.), paliperidone (0.1, 0.5, 1, 2 and 6 mg/kg s.c.) and vehicle (PEG solution, s.c.) [n = 8–9 per group] [36]; each rat received only one dose. Prior to drug treatment, animals were wrapped in a towel to minimize stress and negate the need for anesthesia [59], and a drop of saphenous venous blood was procured with a 25-gauge needle for baseline blood glucose measurement. Animals were then administered the drug, and then 30 minutes later another blood draw was completed to assess the effects of the antipsychotics on fasting glucose levels. Subsequently, animals were administered the glucose challenge (1 g/kg/ml, i.p.) which was followed by repeated sampling of blood glucose readings every 15 minutes for two hours. Glucose levels were measured by handheld glucometer (One Touch Ultra). Animal handlers were blinded to drug treatment.

### Measurement of insulin resistance by HOMA-IR

To determine whether acute treatment with the antipsychotic drugs created a state of insulin resistance, the homeostatic model assessment of insulin resistance (HOMA-IR) was utilized for fasting measurements during the IGTT. Immediately following the measurement of glucose levels at 30 minutes after drug treatment, a saphenous blood draw (200 μl) was performed using heparinized collecting tubes, to obtain plasma for measurement of insulin levels; blood samples were centrifuged (10,000 RPM, 10 Min, 4°C) and plasma samples were stored at -80°C for future analysis. Insulin levels were measured using the ultra-sensitive rat insulin ELISA kit (Crystal Chem Inc., IL, USA). Plasma samples (5μl) were added and analyzed, in duplicate, on 96 well plates. Samples were incubated, followed by repeated washes. The substrate was added and absorbance was measured at 450 nm– 630 nm, as previously [60]. Calibrators were prepared and used to generate a calibration curve. A reference (non-fasted) animal’s plasma added to all plates served as a reference standard; confirming a high intra-plate reliability.

Determination of insulin resistance in rats was accomplished using the homeostatic model assessment of insulin resistance (HOMA-IR) Eq (1) [61]. The product of both the fasting levels of glucose (expressed as mmol/L) and insulin (μU/ml) 30 minutes post-drug administration is divided by a constant of 22.5. Greater insulin resistance is represented via a larger calculated HOMA-IR score.

$$\left(I_{0}\;x\;G_{0}\right)/22.5\;\mathrm{where}\;I_{0}\;\mathrm{and}\;G_{0}\;\mathrm{are}\;\mathrm{fasting}\;\mathrm{insulinemia}\;\mathrm{and}\;\mathrm{glycaemia}.$$ (1)

### Surgical preparations for Hyperinsulinemic-Euglycemic Clamp (HIEC)

Animals were prepared for surgery under isoflurane anesthesia and pre-operative ketoprofen (5 mg/kg, s.c). Heparinized saline-filled polyethylene cannulae (PE50) were inserted into the right common carotid artery and both exterior jugular veins. The arterial cannula was used to sample blood for measurement of glucose levels and venous cannulae were used for the infusion of insulin and dextrose. All cannulae were tunneled subcutaneously to the nape of neck and exteriorized through a small incision. Sutures were applied to all skin openings, and bupivacaine was applied topically to all surgical sites. Rats were allowed to recover for 24 hours prior to the HIEC.

*HIEC Procedures* (see Fig 1B for sequence of experimental procedures) HIEC procedures were performed as previously in our laboratory [62–64]. Rats were fasted overnight (16 ± 2 hours) and habituated to the cage prior to starting the clamp. The two venous cannulae were connected to auxiliary heparinized saline-filled PE50 tubing, directly attached to infusion-only pumps (Harvard Apparatus, Holliston, MA). After a baseline blood glucose reading from the arterial cannula, insulin infusion (3 mU/kg/min) was initiated (t = 0 minutes) and kept running at a constant rate for the entire experiment. Dextrose (50% w/v) infusion commenced at 8 mg/kg/min (0.96 ml/kg/hr) at t = 10 minutes and the glucose infusion rate (GIR) was adjusted as needed, every 10 minutes, to maintain glucose concentrations at 6.0 mmol/L. Euglycemia was determined when three consecutive blood glucose measurements presented 6.0 ± 0.4 mmol/L at the same GIR. Animals then randomly received a single s.c injection of either vehicle or risperidone (1 or 6 mg/kg) or paliperidone (1 or 6 mg/kg) [n = 5–6 per group], and the clamp was continued for 120 minute duration. The sample size of n = 5–6 animals per group is consistent with previously published studies [37,38]. Handlers were blinded to drug treatment.

### Statistical analysis

Metabolic measures obtained from the IGTT were analyzed by one-way analysis of variance (ANOVA), with drug dose as the between group factor. As previously with the IGTT [56,65], glucose data were summed as the area-under-the-curve (AUC) throughout the 120 minute procedure. For the HIEC data, a between-within group analysis was performed, with drug dose (including vehicle) as the between subjects’ factor and change in GIR from baseline as the within factor. Alpha value was set at *p* < 0.05. LSD post-hoc tests were conducted when a main effect or interaction between main effects was significant. Data were analyzed with (IBM SPSS Statistics for Windows, Version 24.0. Armonk, NY: IBM Corp).

## Results

### IGTT

**Fig 2:**
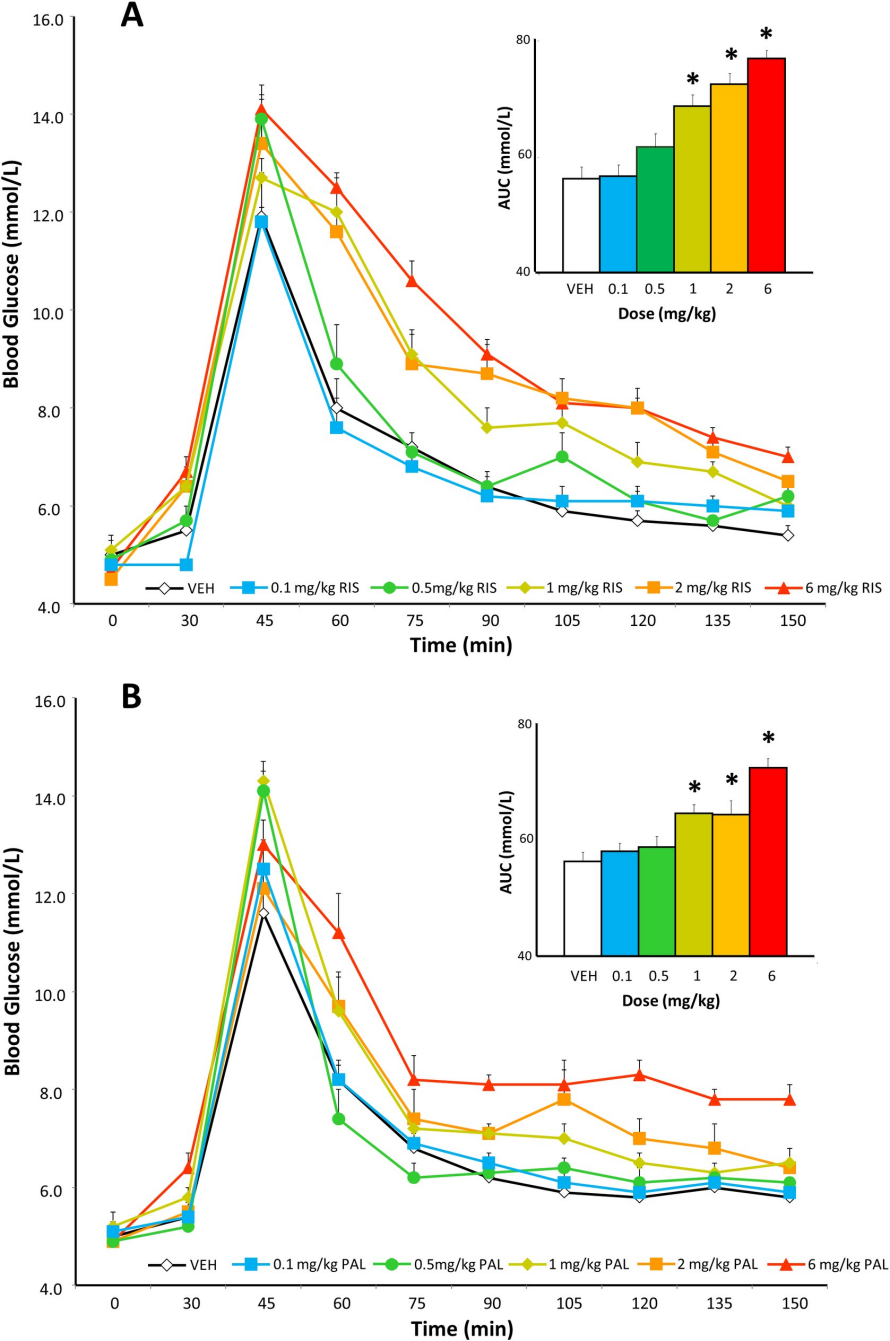
Effects of treatment with the antipsychotic drugs risperidone and paliperidone on fasting glucose levels and during the intraperitoneal glucose tolerance test. Adult female rats (n = 8–9 per group) were administered an acute injection of drug vehicle, (**A**) risperidone (0.1, 0.5, 1, 0.5, 1, 2 or 6 mg/kg, s.c) or (**B**) paliperidone (0.1, 0.5, 1, 0.5, 1, 2 or 6 mg/kg, s.c). Plasma glucose levels were measured before antipsychotic administration in animals fasted overnight at baseline (= Time 0), and then thirty minutes after antipsychotic treatment. Upon completion of this measurement, animals were challenged with the intraperitoneal glucose tolerance test (1 mg/ml/kg of glucose). Plasma glucose levels were recorded every fifteen minutes for a 2 hour period. Cumulative amounts of plasma glucose (group means ± SEM) were added up and presented as the “area under the curve”, evident in the inset figure. Asterix (*) indicates statistically different from control rats, p < 0.05.

The analysis of glucose levels by ANOVA in the IGTT for risperidone-treated rats demonstrated that there were no group differences in pre-treatment baseline glucose levels [F_(5,47)_ = 1.19, NS] (Fig 2A). However, 30 minutes following treatment with the drug (but prior to the glucose infusion), there was a notable effect of drug treatment [F_(5,47)_ = 4.18, p < 0.001]. Post-hoc tests indicated that this represented a dose-dependent effect, whereby the fasting glucose levels were significantly higher than controls for the 1, 2 and 6 mg/kg doses (p < 0.05). Following the glucose challenge, plasma glucose levels rose rapidly in all groups. Comparing the AUCs throughout the 120 minute procedure for glucose concentrations, there was a strong effect of drug treatment [F_(5,47)_ = 20.12, p < 0.001]. Follow-up analyses confirmed a dose-dependent effect on glucose levels, with increasing values for each drug dose. The 0.5 mg/kg dose exhibited a marginally significant increase in AUC glucose levels compared to controls (p = 0.054), while the 1, 2 and 6 mg/kg doses all evinced substantial increases in AUC values (p < 0.001); these effects were evident 15–30 minutes after the glucose challenge.

For the animals treated with paliperidone, the ANOVA indicated again no difference in pre-drug baseline glucose levels [F_(5,49)_ = 0.26, NS] (Fig 2B). Thirty minutes following paliperidone there was a significant effect of drug treatment [F_(5,49)_ = 3.43, p < 0.05], in which post-hoc tests confirmed that the highest (6 mg/kg) dose of paliperidone increased fasting glucose levels significantly compared to controls (p < 0.01). After the glucose challenge, the AUC values displayed an effect of drug treatment [F_(5,49)_ = 11.78, p < 0.001]. The post-hoc tests noted a dose-dependent effect, whereby the 1, 2 and 6 mg/kg doses had significantly greater AUC values than the controls (p < 0.005). The significant increase in glucose levels for these three groups occurred from 30–60 minutes after the glucose challenge.

### HOMA-IR

As described above, plasma samples were obtained 30 minutes after drug treatment and prior to the glucose challenge (Table 1). For the animals treated with risperidone, the analysis of fasting insulin levels with ANOVA noted that there was a significant effect of drug treatment [F_(5,47)_ = 5.61, p < 0.001]. Post-hoc tests confirmed that fasting insulin levels were higher than controls for all groups other than the 0.1 mg/kg dose, where there was a non-significant trend towards significance (p = 0.064). There was also a significant main effect of risperidone treatment on HOMA-IR values [F_(5,47)_ = 8.87, p < 0.001], whereby all but the 0.1 mg/kg group had significantly higher HOMA-IR values (p < 0.005), indicative of whole body insulin resistance.

**Table 1:** Levels of plasma fasting glucose, insulin levels and HOMA-IR values in animals treated with risperidone and paliperidone.

Antipsychotic Drug	Measure	Treatment Dose (mg/kg)					
**Risperidone**		**0**	**0.1**	**0.5**	**1**	**2**	**6**
	**G**_**0**_	5.5 ± 0.2	4.8 ± 0.1	5.7 ± 0.3	6.4 ± 0.4*	6.4 ± 0.3*	6.7 ± 0.3*
	**I**_**0**_	17.8 ± 2.2	25.9 ± 2.4	33.4 ± 4.9*	32.2 ± 3.4*	33.9 ± 6.0*	37.5 ± 1.7*
	**HOMA-IR**	4.3 ± 0.5	5.55 ± 0.5	8.2 ± 1.1*	9.2 ± 1.3*	9.6 ± 1.9*	11.2 ± 0.7*
**Paliperidone**		**0**	**0.1**	**0.5**	**1**	**2**	**6**
	**G**_**0**_	5.4 ± 0.2	5.4 ± 0.1	5.2 ± 0.4	5.8 ± 0.2	5.5 ± 0.2	6.4 ± 0.3*
	**I**_**0**_	14.9 ± 2.0	22.1 ± 2.3	33.8 ± 6.0*	33.9 ± 3.3*	31.3 ± 3.8*	34.8 ± 2.5*
	**HOMA-IR**	3.5 ± 0.5	5.3 ± 0.5	8.1 ± 1.6*	8.9 ± 2.8*	7.8 ± 1.0*	9.9 ± 0.9*

G_0_ = fasting glucose levels (mmol/L); I_0_ = fasting insulin levels (μU/ml); HOMA-IR = Homeostatic Model Assessment of Insulin Resistance (μU**·**mmol) / (ml**·**L). Adult female rats (n = 8–9 per group) were treated with five separate doses of risperidone, paliperidone or vehicle. Values are group means ± SEM. Measurement occurred at t = 30 min of the intraperitoneal glucose tolerance test.

* statistically different from control animals, p < 0.05.

For the paliperidone-treated animals, the ANOVA indicated a main effect of drug treatment on fasting insulin levels [F_(5,49)_ = 5.14, p = 0.001]. Insulin levels were significantly higher in all but the lowest dose (0.1 mg/kg) group compared to controls (p < 0.01). With regards to HOMA-IR values, there was a main effect of drug treatment [F_(5,49)_ = 5.14, p < 0.001]. Post-hoc tests confirmed that HOMA-IR values were larger than controls for all groups other than the 0.1 mg/kg dose (p < 0.005).

### HIEC

Average basal glucose levels were similar for all groups prior to euglycemia and administration of the two antipsychotic drugs. Insulin resistance during the HIEC is inferred by a decrease in the GIR, and therefore the main analysis compared the effects of antipsychotic drug treatment on the GIR.

**Fig 3:**
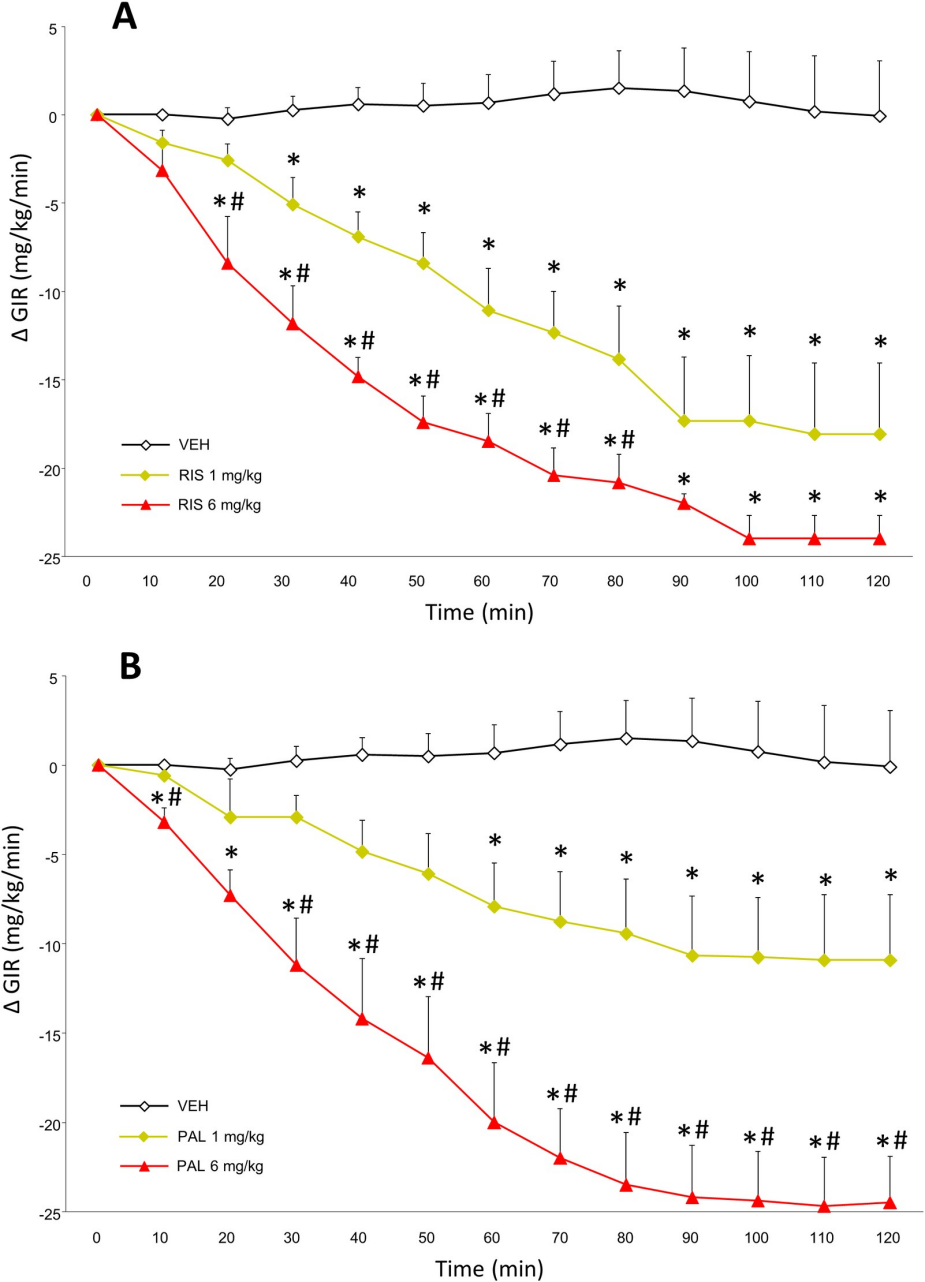
Insulin resistance following the acute administration of the antipsychotic drugs risperidone and paliperidone, measured with the hyperinsulinemic euglycemic clamp. Fasted adult female rats (n = 5–6 per group) were assessed for insulin resistance by the hyperinsulinemic-euglycemic clamp. Animals were stabilized until euglycemia was obtained and then animals were administered vehicle or (**A**) a lower (1 mg/kg) or higher (6 mg/kg) dose of risperidone or (**B**) a lower (1 mg/kg) or higher (6 mg/kg) dose of paliperidone (at t = 0 min). Plasma glucose concentrations were measured every subsequent ten minutes, after which glucose infusion rates (GIRs) were modified if necessary. The GIRs are provided as the change in GIR from euglycemia (group means ± SEM). Asterix (*) represents significantly different compared to controls, p < 0.05; hashtag (#)represents significantly different versus the 1 mg/kg dose-treated animals, p < 0.05.

For the risperidone treated rats, the overall ANOVA indicated significant main effects of both drug treatment [F_(2,15)_ = 28.55, p < 0.0001], time [F_(12,180)_ = 35.69, p < 0.0001] and a drug × time interaction [F_(24,180)_ = 10.42, p < 0.0001]. Post-hoc analysis revealed that both doses of risperidone caused a large decrease in the GIR (Fig 3A), reflecting a clear increase in whole body insulin resistance. Both risperidone doses significantly decreased the GIR compared to control animals (p < 0.001), and the 6 mg/kg dose of risperidone decreased the GIR more so than the 1 mg/kg dose (p < 0.05). Significant decreases in the GIR were evident by 20 minutes after treatment with the 6 mg/kg dose and by 30 minutes with the 1 mg/kg dose of risperidone. Insulin resistance was still near its peak at the end of the 120 minutes duration clamp.

With regards to the paliperidone treated rats, the ANOVA observed a main effect of drug treatment [F_(2,14)_ = 17.21, p < 0.0001], a main effect of time [F_(12,168)_ = 31.13, p < 0.0001] and a drug × time interaction [F_(24,168)_ = 11.66, p < 0.0001]. The post-hoc analysis confirmed that the GIR was significantly different between all three groups (Fig 3B), with both doses of paliperidone exhibiting a lower GIR than controls (p < 0.05), and the 6 mg/kg dose of paliperidone having a lower GIR than the 1 mg/kg dose (p < 0.005). Decreases in the GIR were rapid with the 6 mg/kg dose, with significant reductions compared to both the controls and the 1 mg/kg dose by the first 10 minutes after treatment. For the 1 mg/kg dose of paliperidone, decreases in the GIR occurred more gradually, and did not differ significantly from controls until 60 minutes after drug treatment.

## Discussion

In the present study, we compared how the two closely-related second generation antipsychotic drugs, risperidone and paliperidone, caused acute metabolic side-effects versus vehicle controls, using well-established preclinical models. With a wide range of drug doses, glucose intolerance was assessed by measuring the effects of drug treatment on fasting glucose levels and also following a glucose challenge in the glucose tolerance test, while insulin resistance was initially determined by measuring glucose and insulin levels, and applying the HOMA-IR equation. As the use of HOMA-IR is associated with limitations [66], we followed up using the HIEC, which is the “gold standard” measure of whole body insulin resistance [67]. The overall findings were broadly similar between the two drugs, in that both were associated with fairly substantial metabolic dysregulation. Fasting glucose levels were increased by the three highest doses of risperidone, whereas only the highest dose of paliperidone increased fasting glucose levels. Following the glucose challenge, the three highest doses of both drugs significantly increased glucose intolerance, with a strong trend for the 0.5 mg/kg dose of risperidone to do so, but not with the equivalent dose of paliperidone. These findings suggest that risperidone might have slightly great metabolic liability to cause hyperglycemia than paliperidone. There was no statistical difference between both drugs with regards to insulin resistance based on the HOMA-IR analysis. In the HIEC, both doses of risperidone and paliperidone caused a marked decrease in the GIR, reflecting substantial insulin resistance; in magnitude, this effect was larger for the lower (1 mg/kg) dose of risperidone than the same dose of paliperidone. The current doses of drugs were chosen to be consistent with prior studies that have observed metabolic effects of risperidone in both our laboratory and by other groups [36,37,57], and at preclinical doses comparable to those observed with other antipsychotic drugs which exert effects of a similar magnitude to those noted in humans [45].

The present experimental procedures provide complementary techniques to assess the overall metabolic side-effects of antipsychotic drugs, as we and multiple other groups have demonstrated previously [39–44,68,69]. Fasting glucose levels are the simplest and most common way used to detect hyperglycemia in humans (and animals), but can be prone to error in a significant proportion of individuals [70]. The glucose tolerance test is therefore used as the standard assay to determine glucose intolerance, and is frequently used in both preclinical and clinical studies of metabolic syndrome, prediabetes and Type 2 DM [67]. As insulin and glucose levels are free to change during the glucose tolerance test, it is not suitable for providing an index of insulin sensitivity. The most accurate measure of whole-body insulin resistance is provided by clamp procedures such as the HIEC, which enables the precise assessment of insulin-mediated uptake of glucose [71]. In previous studies, we have noted a general agreement between glycemic dysregulation using both the glucose tolerance tests and the HIEC [62,63], but in some instances there may be changes in one and not the other [64], and they may not be equally sensitive to drug effects.

The observation that risperidone increased fasting glucose levels and caused glucose intolerance is consistent with prior observations from our laboratory, where we previously noted significant glucose intolerance with risperidone at comparable doses [36,57]. Findings are also consistent with an earlier study by Baptista and colleagues [72], whereby lower doses of risperidone (≤ 0.5 mg/kg) did not cause glucose intolerance in female rats, whereas a recent study noted that a higher dose of risperidone (0.9 mg/kg, b.i.d.) increased fasting glucose levels and induced glucose intolerance [73], similar to the current results. We are aware of two prior studies that have assessed the effects of risperidone on insulin sensitivity in rats using the HIEC. In the first of these [37], a 2 mg/kg dose of the drug did not affect the GIR, indicating no effect on insulin sensitivity. In contrast, the more recent study observed that a 1 mg/kg dose of risperidone potently decreased the GIR [38], indicating pronounced insulin resistance, and thus is consistent with our present findings of lowered GIR with the same dose of the drug. It is not immediately clear why the inconsistency between the studies exists–the present study used female rats which are more sensitive to antipsychotic-induced metabolic side-effects [32], although both of the other studies used male rats, leaving the question open as to why the discrepancy exists between those prior reports; further study may be required, but the balance of evidence suggests that risperidone does indeed exert dose-dependent acute metabolic effects. With regards to paliperidone, there is little in the preclinical literature on the metabolic side-effects of the drug, which is somewhat unexpected, due to the widespread use of the antipsychotic in clinical populations [74]. One prior study reported that repeated treatment with a dose of 1.25 mg/kg of paliperidone did not increase fasting glucose levels or HOMA-IR [75]. This stands in partial contrast to the present, where we also observed no effect on fasting glucose levels at this dose, but we noted a pronounced increase in HOMA-IR with doses as low as 0.5 mg/kg. The explanation for this dissociation of effects on fasting glucose versus following a glucose challenge is beyond the scope of the present study, and will require further study to determine. However, clinical studies in humans have observed that different subgroups with prediabetes have demonstrated impairments in fasting glucose but not the tolerance test, and vice versa [76]. This has been interpreted by impaired fasting glucose deficits reflecting selective deficits in basal insulin secretion and glucose-stimulated first-phase insulin secretion, whereas deficits in response to the glucose challenge reflect impaired glucose-stimulated first and second-phase insulin secretion; future studies should be designed to determine whether these mechanisms underlie the metabolic effects of paliperidone. We also observed a significant reduction in the GIR with both the 1 and 6 mg/kg doses of paliperidone in the HIEC. This partial discrepancy may be caused by a difference in sexes used for the studies, as [75] utilized male rats, which a fairly extensive literature indicates are less sensitive to antipsychotic-induced metabolic side-effects [32,33,54], although there are also notable differences in insulin sensitivity between rats strains, including the two strains used in these studies [77]. The reasons for the greater metabolic effects of antipsychotics in female versus male rats remain an ongoing area of study. Experts in the field have noted the impact of antipsychotic drugs on a range of endocrine substrates which differ between males and females, including the sex hormones and prolactin, modification of which (for example, by antidiabetic agents such as metformin) can lead to changes in insulin sensitivity [78,79]. While both male and female human patients show broadly similar metabolic changes with antipsychotic drugs (although see [54]), the metabolic changes observed in female rats treated with antipsychotics indicates that they more accurately model the human condition, and so may represent a better choice for studying the metabolic effects of these drugs.

The modest differences in metabolic effects between risperidone and paliperidone may be due to both pharmacodynamic and pharmacokinetic differences between the two drugs. Even though paliperidone is closely structurally related to its parent compound risperidone (differing only by a hydroxyl group in position 9 of the risperidone molecule), the two compounds differ in their affinity for various central and peripheral receptors which regulate glucose levels. These dissimilarities vary, depending on the conditions of the study [25], but one of the more pronounced differences is a lower receptor affinity by paliperidone for α1 and α2 adrenoceptors versus risperidone in living animals [29,80]. Interestingly, stimulation of α_1_- adrenoceptors increases glucose uptake in muscle and adipose tissue [81,82], and so blockade of the receptor might be expected to increase glucose levels. In parallel, α_2_-adrenoceptors modify the release of multiple hormones associated with glucose regulation [83], as α_2_-adrenoceptor antagonists typically tend to improve metabolic control of glucose [84], suggesting a complex interaction between the two adrenoceptors. Risperidone and paliperidone also differ in their affinity for the 5-HT2A and 5-HT1D receptors [80], which both play an important role in glucose and insulin regulation [85,86]. Pharmacokinetic differences are also evident between the two drugs, as paliperidone exhibits a lower affinity than risperidone for the P-glycoprotein transporter [25,87], which regulates the ability of the drugs to cross the blood brain barrier, and results in greater concentrations in the brain of risperidone than paliperidone [29]. As there is an increasing body of evidence which indicates that some of the metabolic side-effects of antipsychotics may be centrally mediated [88,89], differences in affinity for P-glycoprotein could theoretically affect the magnitude of metabolic changes. The two antipsychotics are also metabolized differently, as risperidone is metabolized in the liver mainly by the polymorphic cytochrome P450 2D6 (CYP2D6) to its active metabolite 9-hydroxyrisperidone (paliperidone) [90]. Paliperidone, by contrast, is predominantly excreted unchanged in the urine [91]. Risperidone has a significantly shorter half life than paliperidone, with the plasma elimination half-life of risperidone and time to maximal concentration being approximately half as long as with paliperidone in rats [58]. This may explain the longer duration required to observe significant reductions in the GIR with the lower dose of paliperidone versus the same dose of risperidone. Clearly, this complex mix of pharmacodynamic and pharmacokinetic differences between risperidone and paliperidone will require further study to determine which contribute to the modest differences in metabolic activity.

Interpretation of the current results is somewhat complicated by the concurrent presence in multiple body organs of both active compounds in the risperidone-treated rats, but only paliperidone for those treated with that drug. Risperidone is quickly metabolized within minutes into paliperidone in rats, where plasma levels of the parent compound and the metabolite remain at approximately equal levels for the first couple of hours [92], consistent with our pilot data (unpublished observations). Thus, at the time of both the glucose tolerance test and the HIEC, the risperidone-treated rats are experiencing the metabolic effects of two active drugs, versus only one drug for the paliperidone-treated rats. However, paliperidone concentrations in most organs in risperidone-treated animals do not achieve the same maximal concentration as with paliperidone-only treatment, as a portion of the risperidone is eliminated directly [93]. To disentangle the specific contribution of each drug as part of the total active moiety in risperidone-treated rats would be challenging, and is beyond the scope of the present study. The goal of the current series of experiments was to directly compare the metabolic effects of equivalent doses of risperidone and paliperidone, as might be observed in the clinic. In comparison to other second generation antipsychotic drugs, the present results suggest that the acute metabolic effects of risperidone and paliperidone tend towards the more metabolically active antipsychotics, rather than the less active ones. Based on studies from our laboratory using similar techniques [62–64,94], the magnitude of the metabolic effects more closely resembles those of drugs such as clozapine, olanzapine and iloperidone, which have greater metabolic liability, versus antipsychotics such as asenapine and lurasidone, which exerted lesser metabolic effects.

In summary, the present results indicate that both risperidone and paliperidone induce glucose dysregulation, with slightly greater effects of the former, depending on the dose and technique used. These findings are in general agreement with the clinical literature. Both drugs have established effects on weight gain and fasting glucose levels in patient populations [21,22,95]. While results vary by study, there is a general tendency for these effects to be slightly greater in magnitude with risperidone than paliperidone, although when analyzed statistically head-to-head these differences are typically not statistically or clinically significant [96]. Additional comparative studies of the metabolic side-effects of these drugs in clinical subjects using more detailed procedures, such as the glucose tolerance test and the HIEC, may help determine whether both drugs differ in their metabolic liability. Given the large number of patients who are currently treated with both antipsychotics, for what are often life-long conditions, even slight differences in metabolic dysregulation may have cumulative effects, and so more thorough metabolic evaluations of the drugs in humans would seem warranted.

## Supporting information

S1 File (XLSX)

S2 File (XLSX)

S3 File (XLSX)
